# Polyphenol-Rich Extract from *Archidendron clypearia*: Optimization, Characterization, and Hypouricemic Activity

**DOI:** 10.3390/molecules31091451

**Published:** 2026-04-27

**Authors:** Danna Yan, Ziyan Hong, Zhimin Zhao, Wenzhe Yang, Depo Yang

**Affiliations:** 1 School of Pharmaceutical Sciences, Sun Yat-sen University, Guangzhou 510006, China; yandn@mail2.sysu.edu.cn (D.Y.); hongziyansysu@163.com (Z.H.); zhaozhm2@mail.sysu.edu.cn (Z.Z.); 2 Zhongshan Unicare Natural Medicine Co., Ltd., Zhongshan 528437, China; fabrice0409@163.com

**Keywords:** *Archidendron clypearia*, ultrasonic-assisted extraction, polyphenols, hyperuricemia, xanthine oxidase, urate transporters

## Abstract

This study aimed to optimize the ultrasonic-assisted extraction of polyphenols from *Archidendron clypearia* and to evaluate their anti-hyperuricemic effects. Polyphenols from medicinal plants have attracted increasing attention due to their potential roles in regulating uric acid metabolism. In this study, single-factor experiments combined with Box–Behnken response surface methodology were employed to optimize extraction conditions, and an entropy weighting method was applied to integrate total polyphenols and Archidendrin I into a comprehensive evaluation index. The bioactivity of the obtained extract was further assessed through in vitro assays and a hyperuricemic mouse model. The optimal extraction conditions were determined to be 50% ethanol, a liquid-to-material ratio of 30, and 31 min of sonication, yielding 175 mg GAE/g DW of total polyphenols and 80.34 mg/g DW of Archidendrin I. The extract exhibited significant xanthine oxidase inhibitory activity, reduced serum uric acid levels, regulated urate transporters (URAT1, GLUT9, and ABCG2), and alleviated renal and hepatic injury in hyperuricemic mice. These findings indicate that the optimized process enables efficient extraction of polyphenols from *Archidendron clypearia*, and the resulting extract exerts beneficial regulatory effects on uric acid metabolism, highlighting its potential as a natural agent for hyperuricemia management.

## 1. Introduction

Hyperuricemia (HUA), characterized by elevated serum uric acid (UA), is a prevalent metabolic disorder and a key risk factor for gout and chronic kidney disease [1]. UA homeostasis is governed by its production via XOD and renal excretion regulated by transporters including URAT1 (reabsorption) and ABCG2 (excretion) [2]. Simultaneously modulating both pathways represents a promising therapeutic strategy for HUA. Current first-line drugs, such as xanthine oxidase (XOD) inhibitors (e.g., allopurinol) and uricosuric agents (e.g., benzbromarone), primarily act on single targets and are associated with adverse effects [3]. For instance, allopurinol may cause severe hypersensitivity reactions, including Stevens–Johnson syndrome [4], as well as hepatotoxicity and renal impairment [5]. Febuxostat has been reported to increase the risk of cardiovascular events [6]. Uricosuric agents such as benzbromarone may induce hepatotoxicity and increase the risk of uric acid nephrolithiasis [7,8]. These limitations highlight the need for safer and multi-target therapeutic agents, particularly those derived from natural products, driving the search for safer, multi-target alternatives from natural products.

*Archidendron clypearia* (Jack) I. C. Nielsen is a plant species belonging to the Fabaceae family. It is widely distributed across southern China and various tropical regions in Asia. *Archidendron clypearia* is a characteristic medicinal plant in traditional Chinese medicine from southern China, with the functions of clearing heat and detoxifying, cooling the blood, and reducing swelling. It has been traditionally used for the treatment of tonsillitis, gastric pain, and damp-heat diarrhea. Modern pharmacological studies have shown that *A. clypearia* exhibits anti-inflammatory, antioxidant, antiviral, and antibacterial activities [9,10], earning it the reputation of a “green antibiotic”. Its major chemical constituents include flavonoids, phenolic acid, phenylpropanoids, triterpenoids, and steroids [9,11,12]. Among these, flavonoids and organic acids, collectively referred to as polyphenols, constitute the predominant portion of its bioactive compounds. These polyphenolic components are considered the primary active substances responsible for the herb’s medicinal effects. Archidendrin I (also known as 7-O-galloyltricetiflavan) is one of the most abundant compounds in *A. clypearia* and exhibits superior antioxidant activity, which is about 2000 times higher than that of Trolox and substantially exceeds those of trans-resveratrol and ascorbic acid [13]. An in vitro study evaluating xanthine oxidase inhibition has demonstrated the superior inhibitory activity of Archidendrin I, suggesting its potential for uric acid-lowering effects [14]. However, despite its promising bioactivities, standardized polyphenolic extracts of *A. clypearia* are lacking, and their anti-hyperuricemic effects and underlying mechanisms, particularly in relation to uric acid metabolism, remain poorly understood.

Based on the above, this study developed, for the first time, an optimized extraction method for polyphenolic constituents from *Archidendron clypearia*, using total phenolic content and representative components as evaluation indicators. The resulting extract was designated as APE2025 (*Archidendron clypearia* polyphenol extract), where “APE” denotes the extract type and “2025” indicates the year in which the extraction process was established.

APE2025 was subsequently evaluated both in vitro and in vivo for its potential effects on hyperuricemia. The results indicate that APE2025 can inhibit uric acid production, regulate uric acid excretion, and alleviate renal injury in hyperuricemic mice. To our knowledge, this study represents one of the early systematic investigations into the uric acid-lowering effects of polyphenol-rich extracts from *A. clypearia* and their possible mechanisms. These findings provide experimental support for the further exploration of APE2025 as a natural product candidate for hyperuricemia management.

## 2. Results

### 2.1. Extraction Optimization

#### 2.1.1. Single-Factor Test

**Figure 1:**
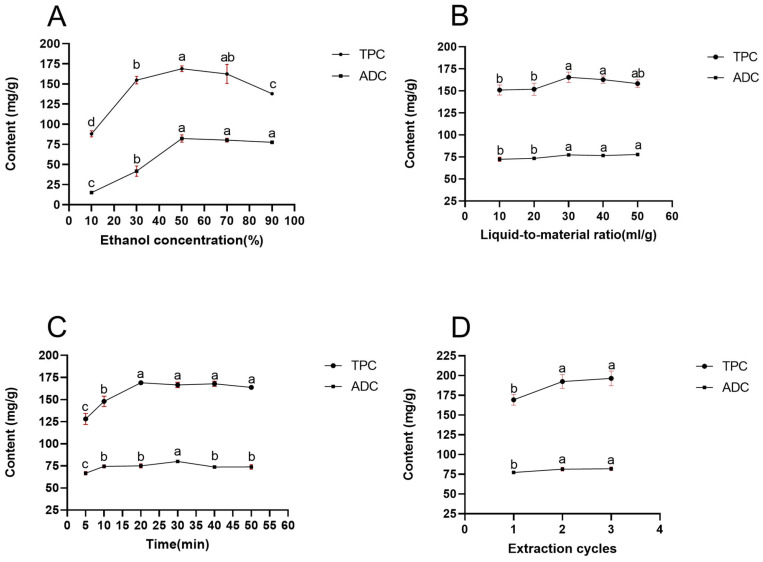
Effect of different factors on extraction rate of total polyphenols and Archidendrin I. (**A**) effect of ethanol concentration; (**B**) effect of liquid-to-material ratio; (**C**) effect of extraction time; (**D**) effect of extraction cycles. Different lowercase letters indicate significant differences among groups at *p* < 0.05 based on one-way ANOVA.

The effect of each factor on extraction rate was investigated with single-factor tests. Results indicated that the extraction efficiency was affected by ethanol concentration, liquid-to-material ratio, ultrasonic time and extraction cycles (Figure 1).

Methanol and ethanol, as polar solvents, are commonly employed for the extraction of polyphenolic compounds from plant samples [15,16]. Moreover, ethanol is recognized for its safety and environmental friendliness, which justifies the selection of ethanol-water mixtures as the solvent in this study. As shown in Figure 1A, when the ethanol concentration increased from 10% to 50%, TPC (total polyphenol content) and ADC (Archidendrin I content) increased accordingly, reaching their maximum extraction yields at 50% ethanol. According to the principle of “like dissolves like,” the solubility of the target compounds is highest under these conditions, while a higher ethanol concentration corresponds to lower solvent polarity. When the ethanol concentration deviated from 50%, either higher or lower, the extraction efficiency of the bioactive compounds decreased. Our results show that the extraction of total phenolics and Archidendrin I from *Archidendron clypearia* is optimal at an ethanol concentration of 50%; therefore, 50% ethanol solution was selected for subsequent experiments.

Figure 1B shows that with the gradual increase in the liquid-to-material ratio, the extraction yield of polyphenols initially rose alongside the solvent volume, eventually leveling off. This trend can be attributed to the enhanced wettability of the powder and the expanded contact area between the liquid and material as more solvent was used. An increase in concentration difference between the inner and outer cells contributed to the dissolution of the active substance. However, when the liquid-to-material ratio exceeded 20 for TPC and 30 for ADC, the contribution of this factor to the extraction yield became negligible. At this stage, most target compounds had been extracted, and further increases in solvent volume would only result in resource waste. Therefore, based on a comprehensive evaluation of extraction efficiency and economic principles, a liquid-to-material ratio of 30 was selected for subsequent experiments.

Extraction time plays a crucial role in the extraction process. In this study, the sonication time was varied from 5 to 50 min while keeping other parameters constant. As shown in Figure 1C, the total polyphenol content (TPC) increased with prolonged extraction time from 5 to 20 min, reaching its maximum at 20 min. No significant differences in TPC were observed among the time points of 20, 30, 40, and 50 min (not) based on analysis of variance (ANOVA). In contrast, the Archidendrin I content (ADC) peaked at 30 min and subsequently declined. These phenomena may be attributed to the intense mechanical shear effects generated by ultrasonication, which can lead to structural degradation of polyphenols, increased dissolution of impurities, and a reduction in active compounds [17]. Considering both extraction efficiency and economic feasibility, 30 min was selected as the optimal extraction time.

Regarding extraction cycles, the polyphenols in *Archidendron clypearia* were almost completely extracted after two cycles. No significant difference was observed in the yields of TPC or ADC between two and three extraction cycles. Therefore, two extraction cycles were selected as the optimal condition.

The reliability of the analytical method was confirmed by good linearity of calibration curves (Figures S1 and S2) and well-resolved chromatograms (Figure S3).

#### 2.1.2. Box–Behnken Design

Significant impacts of the three selected factors on both TPC and ADC were observed in the preliminary single-factor tests. Informed by these results, a Box–Behnken (B-B) experimental design with three factors and three levels was subsequently constructed. Refer to Table S3 for the designated levels of each factor. Objective weighting of the two response variables was achieved via the entropy weight method (EWM), which assigned weights of 0.4706 to TPC and 0.5294 to ADC. For each experimental run, a combined score, termed the comprehensive evaluation value (CEV), was computed as per the equation: CEV = 0.4706 × TPC + 0.5294 × ADC. The detailed experimental arrangement and outcomes are compiled in Table 1.

**Figure 2:**
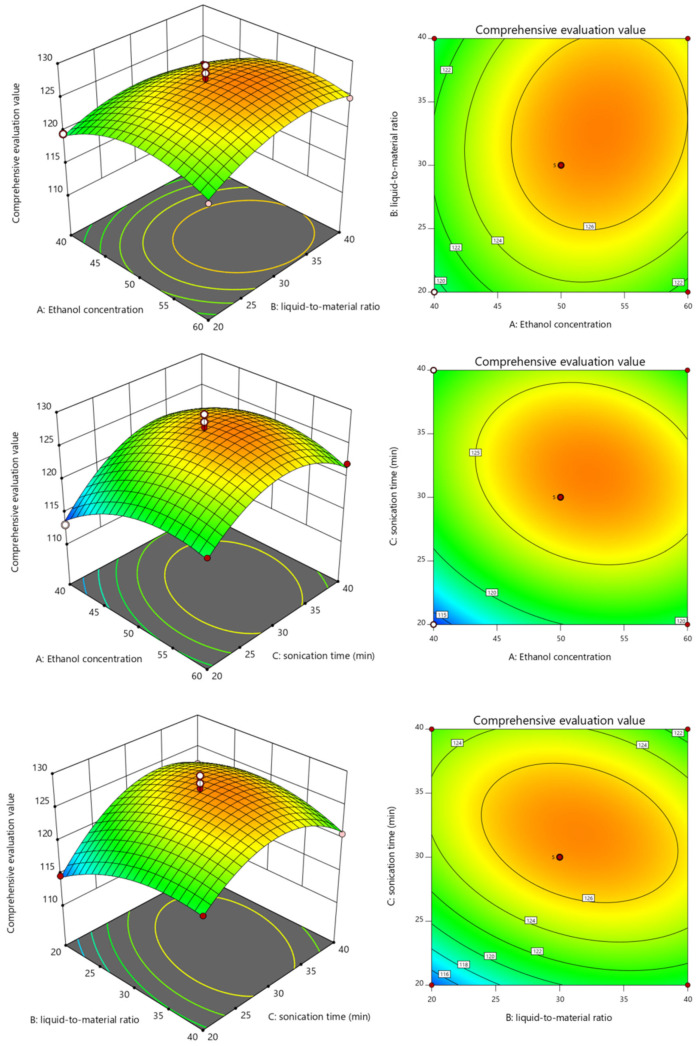
Three-dimensional response surface diagrams of the influence of different factors on the composite evaluation value.

The response surface experimental design in Table 1 was completed using Design-Expert 11 software. Analysis of variance (ANOVA) was applied in the model, and the following equation was obtained after multiple quadratic regression: CEV = 127.09 + 1.71A + 1.13B + 2.18C + 0.7204AB − 1.34AC − 1.84BC − 3.15A^2^ − 2.51B^2^ − 4.99C^2^ (R^2^ = 0.9363). The ANOVA results are presented in Table 2. The difference between Adj R^2^ (0.8545) and Pred R^2^ (0.8157) indicates reasonable consistency between them, as the difference is < 0.2. With *p* = 0.002 < 0.01, the quadratic regression model is highly significant. The lack-of-fit term (*p* = 0.9339) is not significant, indicating that the model fits the experimental data well and can be used to predict and analyze the results of TPC and ADC experiments in *Archidendron clypearia* under different reaction conditions. The F-values from the ANOVA results for the terms of the regression model equation show that the order of influence of the factors is: sonication time > ethanol concentration > liquid-to-material ratio, with their interaction effects not being significant. Among them, ethanol concentration and ultrasonic time have significant effects on the comprehensive index content, whereas the liquid-to-material ratio does not. To visualize the analytical results, three-dimensional response surface plots were generated using Design-Expert software, as shown in Figure 2. The 3D plots also reveal that ethanol concentration first increases and then decreases, the liquid-to-material ratio first increases and then tends to stabilize, and time first increases and then decreases. Moreover, the variation trend of the liquid-to-material ratio is the smallest, which is consistent with the F-value results in Table 2.

The optimal conditions for the extraction process were determined as A = 52.656, B = 32.101, and C = 31.434, with a corresponding comprehensive evaluation value (CEV) of 127.588. For practical application, these parameters were adjusted to A = 50, B = 30, and C = 31. Under the adjusted conditions, three independent validation experiments were performed. The average CEV obtained was 124.85, with a relative standard deviation (RSD) of less than 3%. The deviation between the experimental average and the model-predicted value was 1.88%. These results demonstrate that the extraction process exhibits good reproducibility and operability.

### 2.2. Qualitative and Quantitative Analysis of APE2025

**Figure 3:**
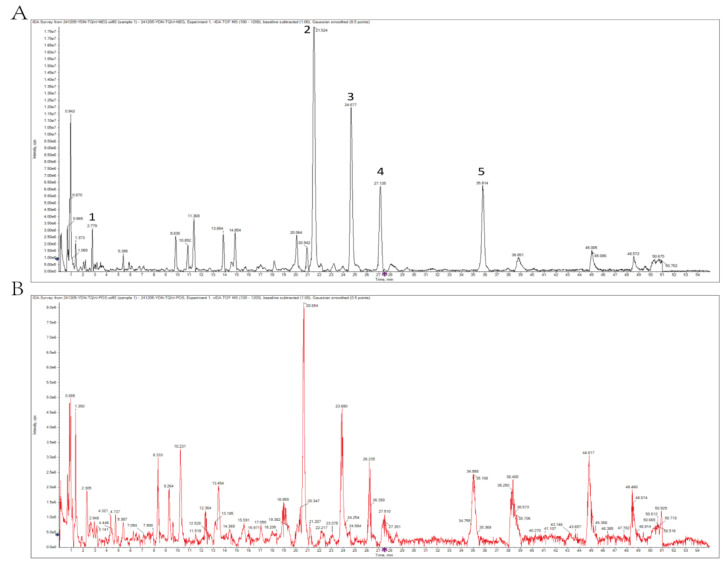
Total ion chromatograms of APE2025 in negative and positive ion modes. (**A**) Negative ion mode; (**B**) positive ion mode. Numbered peaks correspond to compounds listed in Table 3 and Table 4. The blue arrow and purple markers are instrument-generated and do not affect data interpretation.

The optimized process yielded APE2025 with a high content of total polyphenols (45.48%) and Archidendrin I (19.69%), indicating effective enrichment of bioactive constituents. Qualitative analysis of the main constituents was performed by UPLC-Q-TOF-MS. Data processing was carried out using the SCIX OS data processing system. Accurate relative molecular masses were determined from primary mass spectrometry data, while fragment information was obtained from secondary mass spectrometry. By comprehensively analyzing chromatographic retention times and mass spectrometric fragmentation patterns and by comparing them with literature and database references, a total of 23 chemical components were identified. These compounds are listed in Table 3 and Table 4, which present their retention times, precursor ion information, and fragment ion peak data. Five peaks are labeled in Figure 3. Among them, gallic acid and quercitrin were selected as reference compounds due to their established use as quality markers in previous studies, while the remaining peaks were chosen based on their relatively high peak areas in the chromatogram. Most of them are flavonoids and phenolic acids, which are known for their antioxidant and metabolic regulatory activities. The chemical profile suggests that APE2025 is a complex mixture of polyphenolic compounds rather than a single active ingredient, which may contribute to its potential multi-target biological effects.

### 2.3. Xanthine Oxidase Inhibitory Effect of APE2025

**Figure 4:**
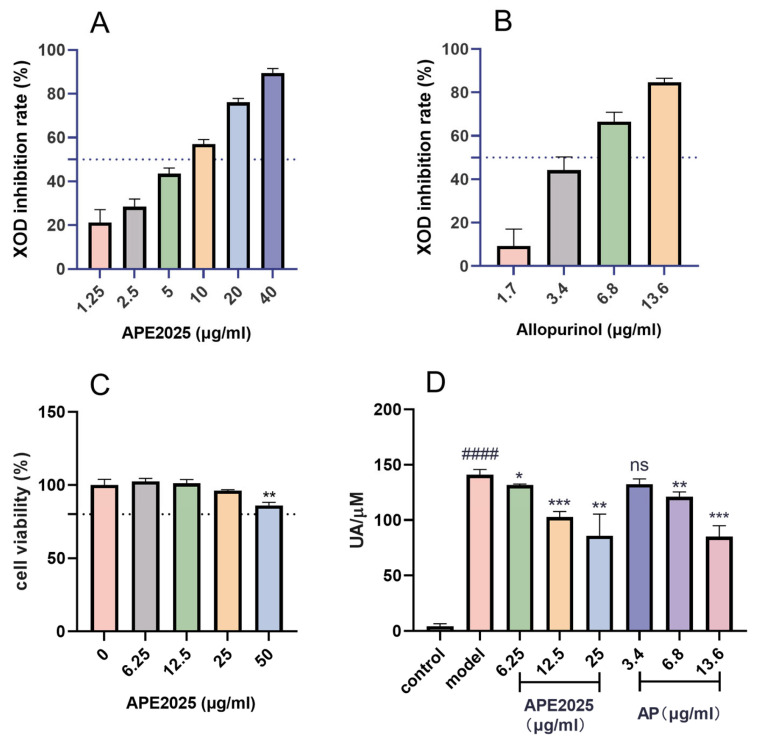
In vitro XOD inhibitory activity and anti-hyperuricemic cellular activity of APE2025. (**A**) APE2025 significantly inhibited XOD activity in vitro; (**B**) Allopurinol suppressed XOD activity; (**C**) Cytotoxicity evaluation of APE2025 in HK-2 cells; (**D**) APE2025 and allopurinol reduced uric acid levels in hyperuricemic cells. The dotted lines in (**A**,**B**) represent 50% inhibition, and the dashed line in (**C**) represents 80% cell viability. #### *p* < 0.0001 vs. the control group; ns *p* > 0.05, * *p* < 0.05, ** *p* < 0.01,*** *p* < 0.01 vs. the model group.

Xanthine oxidase (XOD) is a key enzyme in uric acid production. Excessive XOD activity directly raises serum uric acid, making it a primary target for urate-lowering drugs such as allopurinol and febuxostat. Therefore, inhibiting XOD is a crucial therapeutic strategy for hyperuricemia [2]. We conducted in vitro experiments to evaluate the inhibitory potential of APE2025 against XOD activity. The XOD inhibition rate of APE2025 increased gradually with the concentration (Figure 4A,B), indicating a typical dose–response relationship. At 40.00 µg/mL, APE2025 showed significant activity with an inhibition rate of 89.52%. Its IC_50_ was determined to be 7.11 µg/mL. The gradual increase in inhibition with concentration, followed by an apparent approach to maximal effect, indicates a tendency toward saturation at higher concentrations.

Allopurinol, at 100.00 µM (13.61 µg/mL), exhibited an inhibition rate of 84.67%, with an IC_50_ of 34.05 µM (equivalent to 4.63 µg/mL), which is slightly lower than that of APE2025. Notably, although the IC_50_ value of APE2025 was slightly higher than that of allopurinol, it remained substantially lower than those of several commonly used traditional Chinese medicines for hyperuricemia, such as the extract of Plantago asiatica (IC_50_ = 89.14 µg/mL) [18]. This suggests that APE2025 may achieve broader inhibitory effects at lower concentrations. Such activity may be attributed to the combined action of multiple polyphenolic constituents rather than a single compound.

### 2.4. Anti-Hyperuricemia Effect of APE2025

A cellular model of hyperuricemia (HUA) was established using adenosine and xanthine oxidase (XOD). Compared with the control group, this model exhibited a significant increase in uric acid levels (Figure 4D). The cytotoxicity of APE2025 was evaluated using the CCK-8 assay (Figure 4C). Test concentrations ranged from 50 µg/mL to 6.25 µg/mL. Compared with the control group, HK-2 cells treated with APE2025 showed no significant cytotoxicity at concentrations up to 25 µg/mL. A sharp decline in cell viability was observed at 50 µg/mL, as shown in the figure. Therefore, the highest non-cytotoxic concentration of APE2025 was determined to be 25 µg/mL.

In the hyperuricemia cell model, three concentrations of APE2025 reduced uric acid level in a concentration-dependent manner, as shown in Figure 4. In particular, compared with the model group (140.85 µM), 25 µg/mL of APE2025 significantly decreased UA levels to 85.92 µM (*p* < 0.01). Each incremental increase in concentration resulted in a further decrease in UA levels, demonstrating a clear dose–response relationship. Notably, the reduction between medium (12.5 µg/mL) and high (25 µg/mL) concentrations was less pronounced, suggesting that the effect may begin to plateau at higher concentrations. The allopurinol group also showed a similar concentration-dependent trend, with 13.8 µg/mL of allopurinol (AP) reducing UA levels to 85.10 µM (*p* < 0.001). This suggests that APE2025 effectively suppresses uric acid accumulation at the cellular level. Combined with its XOD inhibitory activity, these results indicate that APE2025 may act by limiting uric acid production.

### 2.5. APE2025 Decreased the Levels of UA and CRE in Serum, and Improved Kidney Coefficient

**Figure 5:**
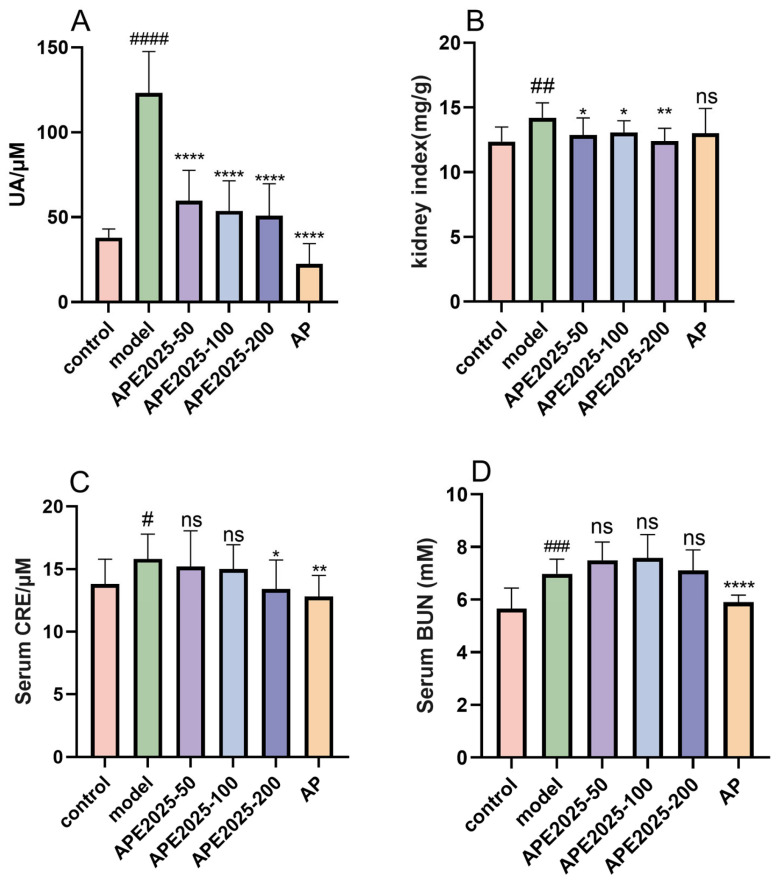
Effect of APE2025 on serum uric acid, creatinine, blood urea nitrogen, and kidney coefficient in hyperuricemic mice. (**A**) The level of serum UA; (**B**) kidney index; (**C**) the level of serum CRE; (**D**) the level of serum BUN. Data are shown as mean  ±  S.E.M of 10 mice in each group. # *p* < 0.05, ## *p* < 0.01, ### *p* < 0.001, #### *p* < 0.0001 vs. the control group; ns *p* > 0.05, * *p* < 0.05, ** *p* < 0.01, **** *p* < 0.0001 vs. the model group.

As shown in Figure 5A, serum uric acid (UA) levels in the model group were significantly increased by 225% compared with the control group (*p* < 0.01). APE2025 treatment significantly reversed the elevated serum UA levels in hyperuricemic mice (*p* < 0.0001). Specifically, compared with the model group, serum UA levels decreased by 51.5%, 56.5%, and 58.7% in the APE2025-50, APE2025-100, and APE2025-200 groups, respectively. The inhibition rates among all groups were relatively close. The incremental reduction from medium to high dose was relatively modest, suggesting that the hypouricemic effect may approach a plateau at higher doses. Compared with the commonly used Chinese herbal medicine Polygonum cuspidatum extract (approximately 54% reduction), APE2025 demonstrates comparable efficacy at lower doses. Allopurinol (AP) intervention also reduced serum UA levels in Hua mice by 81%. Although the reduction was less pronounced than that obtained with allopurinol, APE2025 demonstrated additional benefits in restoring the kidney coefficient, which was not observed in the allopurinol group.

Creatinine (CRE) and blood urea nitrogen (BUN) are important indicators of renal injury. We found that CRE and BUN levels were significantly elevated in the model group (*p* < 0.05 or *p* < 0.001, Figure 5C,D). Both APE2025 and allopurinol treatments significantly inhibited the PO- and HX-induced increase in CRE (*p* < 0.05). However, no significant effect was observed on BUN levels, suggesting that its renal protective effects may be selective rather than comprehensive. These findings indicate that APE2025 not only reduces uric acid levels but also provides partial protection against renal dysfunction, highlighting a potential advantage over conventional urate-lowering drugs.

### 2.6. APE2025 Decreased the Level of XOD in Liver and the Levels of ADA in Serum

Besides xanthine oxidase (XOD), adenosine deaminase (ADA) also plays a key hydrolytic role in human purine nucleotide catabolism. It catalyzes the degradation of adenosine to inosine, which is then converted into hypoxanthine by nucleoside phosphorylase. Hypoxanthine is ultimately oxidized into uric acid, the final metabolic product [19]. Since APE2025 significantly reduced serum uric acid and creatinine levels, we subsequently evaluated its effect on the expression of proteins involved in urate production to further explore the mechanism of action of this extract.

**Figure 6:**
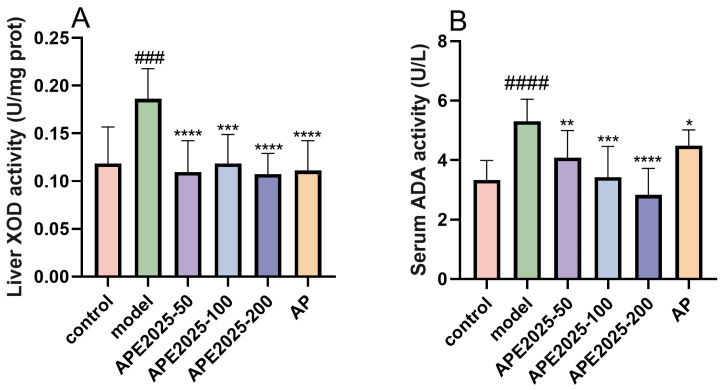
Effect of APE2025 on liver XOD activity and serum ADA activity in hyperuricemic mice. (**A**) The activity of liver XOD; (**B**) the activity of serum ADA. Data are shown as mean  ±  S.E.M of 10 mice in each group. ### *p* < 0.001, #### *p* < 0.0001 vs. the control group; * *p* < 0.05, ** *p* < 0.01, *** *p* < 0.001, **** *p* < 0.0001 vs. the model group.

As shown in Figure 6, the liver XOD level in the model group was 57.45% higher than that in the control group (*p* < 0.001). Treatment with APE2025 significantly reduced liver XOD levels. APE2025-50, APE2025-100, and APE2025-200 treatments restored liver XOD levels in hyperuricemic mice to near-normal levels, showing significant reductions of 41.3%, 36.4%, and 42.38%, respectively, compared with the model group. All three dose groups showed marked reductions compared with the model group, with higher doses generally producing stronger inhibitory effects. These effects were similar to those of allopurinol treatment (40.41% reduction). Figure 6B illustrates that serum ADA levels in the model group were 59.18% higher than those in the control group (*p* < 0.0001). As expected, low-, medium-, and high-dose APE2025 treatments significantly reduced serum ADA levels by 23.08%, 35.38%, and 46.67%, respectively, compared with the model group. This indicates that APE2025 exerts progressively stronger inhibition on ADA with increasing dosage. In contrast, allopurinol treatment only reduced ADA levels by 15.38%. The simultaneous inhibition of these two enzymes suggests that APE2025 may suppress uric acid production at multiple steps in purine metabolism. Notably, APE2025 showed a stronger inhibitory effect on ADA compared to allopurinol, which primarily targets XOD. This indicates that APE2025 may exert a broader regulatory effect on uric acid synthesis pathways, supporting a multi-target mechanism.

### 2.7. APE2025 Improved Kidney and Liver Histopathological Changes in Mice

**Figure 7:**
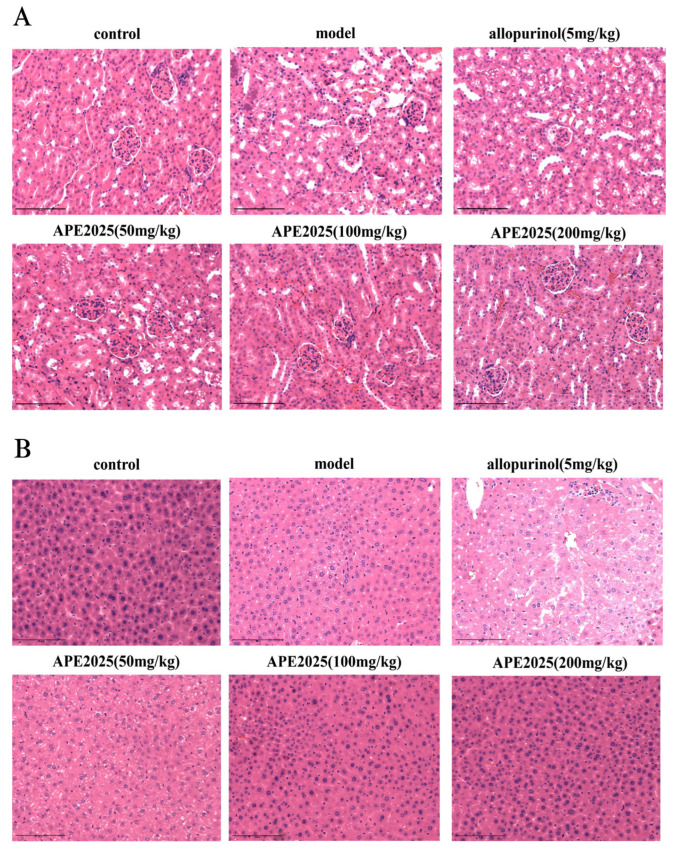
Effect of APE2025 on kidney and liver pathological changes for hyperuricemic mice. (**A**) The representative pictures of kidney histopathology (200×). (**B**) The representative pictures of liver histopathology (200×). Scale bar: 125 μm.

Hyperuricemia frequently leads to structural damage in renal tissues, impairing kidney function through multiple pathways, such as inducing renal inflammation and oxidative stress, damaging renal cortical cells, and promoting renal interstitial fibrosis [1]. H&E staining results showed that in the control group, glomerular boundaries were clear, renal tubules were intact, and no inflammatory cell infiltration was observed (Figure 7A). As shown in the figure, compared with the control group, mice in the hyperuricemia model (HUA) group exhibited glomerular atrophy, tubular dilation, abnormal boundaries between tubular cells, swelling, and mild inflammatory cell aggregation. Allopurinol did not show significant improvement in renal injury repair. In contrast, APE2025 treatment markedly ameliorated morphological lesions in a dose-dependent manner. Renal tubules and glomerular cells appeared relatively clear, with cytoplasmic morphology similar to that of the normal control group, particularly at medium and high doses.

Histopathological examination of liver tissue with H&E staining revealed that in the normal group, hepatocytes were uniform in size and regular in morphology. In the model group, mild pathological injury was observed in the liver tissue, characterized by loose cytoplasm and cellular swelling, presenting a pale-staining appearance (Figure 7B). Compared with the model group, the allopurinol group exhibited more severe hepatic tissue damage, including granular degeneration of hepatocytes, cytoplasmic loosening, and focal infiltration of a small number of inflammatory cells. In the APE2025-treated groups, the medium- and high-dose groups showed significant improvement in liver injury compared to the model group. In the low-dose treatment group, a small number of round microvesicles were still visible within the cytoplasm of hepatocytes, along with loose and pale-staining cytoplasm. Hepatocytes in the medium- and high-dose groups appeared uniform in size and regular in morphology, arranged in an orderly manner without abnormal expansion or compression, similarly to the normal control group. While mild pathological features persisted in the low-dose group, the medium- and high-dose groups exhibited substantially improved cellular morphology, suggesting enhanced hepatoprotective effects at higher doses.

These findings indicate that APE2025 markedly alleviated kidney and liver damage induced by hyperuricemia. In contrast to allopurinol, which showed limited or even adverse effects on liver tissue, APE2025 improved tissue morphology in a dose-dependent manner. These findings suggest that APE2025 possesses organ-protective properties in addition to its hypouricemic effects, which may enhance its therapeutic potential.

### 2.8. APE2025 Restores Expression Levels of Uric Acid Transporter Genes

Uric acid transporters play a critical regulatory role in the absorption and excretion of uric acid in the kidneys and intestines. Abnormal expression of these transporters represents one of the key mechanisms underlying the development and persistence of hyperuricemia [20]. Therefore, a comprehensive analysis of mRNA expression levels of UA transporters URAT1, GLUT9, and ABCG2 was conducted to assess their renal excretion capacity.

**Figure 8:**
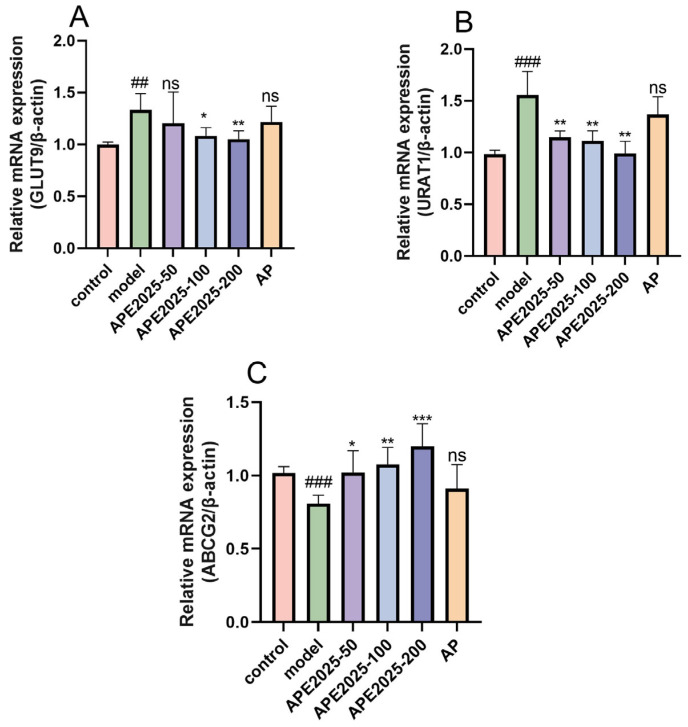
Effect of APE2025 on the mRNA expression of UA transporter proteins. (**A**) The expression of GLUT9; (**B**) the expression of URAT1; (**C**) the expression of ABCG2. Data are shown as mean  ±  S.E.M of 5 mice in each group. ## *p* < 0.01, ### *p* < 0.001 vs. the control group; * *p* < 0.05, ** *p* < 0.01,*** *p* < 0.001 vs. the model group. ns means not significant vs. the model group.

As shown in the Figure 8, mRNA levels of uric acid absorption transporters URAT1 and GLUT9 in renal tissue were elevated in the model group, while mRNA expression of the excretion-related protein ABCG2 was reduced. These findings align with previous studies [21,22], suggesting enhanced uric acid reabsorption and diminished renal uric acid secretion in hyperuricemic mice. Compared with the model group, treatment of HUA mice with APE2025 significantly reduced mRNA expression of URAT1 and GLUT9 while increasing mRNA expression of ABCG2 (*p* < 0.05 or *p* < 0.01). The expression levels of URAT1 and GLUT9 decreased progressively with increasing doses, while ABCG2 expression showed a corresponding increase. This coordinated regulation indicates that higher doses of APE2025 more effectively suppress uric acid reabsorption while enhancing excretion. The graded response across doses suggests a consistent pharmacological effect on urate transport pathways. In contrast, allopurinol did not exhibit these effects. These results indicate that APE2025 lowers uric acid levels by downregulating GLUT9 and URAT1 expression to reduce uric acid reabsorption and upregulating ABCG2 expression to enhance uric acid excretion.

APE2025 significantly downregulated the expression of URAT1 and GLUT9 while upregulating ABCG2. This coordinated regulation indicates that APE2025 reduces uric acid reabsorption and enhances its excretion. Unlike allopurinol, which primarily inhibits uric acid production, APE2025 simultaneously modulates urate transport pathways. This dual regulatory effect suggests a more comprehensive mechanism for restoring uric acid homeostasis.

## 3. Discussion

*Archidendron clypearia* has recently been recognized as a medicinal material with notable therapeutic efficacy. Previous research on this plant has mainly focused on its total polyphenol or extracts, and current quality standards for its preparations only involve the detection of gallic acid and quercitrin. However, Archidendrin I, which is the most abundant component in *Archidendron clypearia*, exhibits excellent antioxidant and antimicrobial activities [13]. Yet, this class of compounds has not received adequate attention and has not been targeted as a specific lead component in process development. Although Archidendrin I has been reported as a major constituent with significant xanthine oxidase inhibitory activity in vitro, it has never been utilized as a key quality indicator for extraction optimization. By integrating the quantification of this component into the Response Surface Methodology (RSM) design, we have moved beyond the conventional “total polyphenol” approach and introduced a compound-specific optimization strategy, thereby linking extraction conditions with pharmacologically relevant composition. This ensures that the resulting extract is not only rich in polyphenols but also enriched with a compound that has a demonstrated relevance to the uric acid reduction. Therefore, extraction optimization in this study was designed not merely to improve yield but to enrich functionally relevant constituents associated with biological activity.

In this study, we systematically optimized the extraction of polyphenols from *Archidendron clypearia*. Compared with conventional single-indicator evaluation, the development and validation of the HPLC-DAD method for quantifying Archidendrin I in parallel with the Folin–Ciocalteu assay for total phenolics represent a more comprehensive and functionally relevant approach for quality assessment. This dual-indicator approach allows for a more nuanced evaluation of extraction efficiency, balancing overall polyphenol recovery with the specific yield of a therapeutically promising constituent [23]. Furthermore, the application of the entropy weight method (EWM) to assign objective weights to these two indicators (TPC and ADC) eliminated subjective bias in process optimization, ensuring that the final optimized conditions (50% ethanol, liquid-to-material ratio of 30, 31 min sonication) reflect a scientifically sound compromise between general extraction efficiency and targeted compound recovery. Importantly, the optimized conditions resulted in the simultaneous enrichment of TPC and ADC, establishing a direct link between extraction parameters and compositional changes. In particular, Archidendrin I, as a major enriched constituent with reported XOD inhibitory activity, is likely to contribute significantly to the observed pharmacological effects.

Hyperuricemia (HUA) has become a prevalent metabolic disorder with a steadily increasing global incidence, posing a significant burden on public health. Although various drugs are available to lower uric acid levels—including uricosuric agents and uric acid synthesis inhibitors—most primarily act through a single mechanism, making it difficult to comprehensively address the complex metabolic disturbances underlying the disease. Furthermore, these medications may present limitations such as a tendency for relapse after discontinuation, adverse side effects, drug interactions, and specific contraindications [24,25]. In recent years, Chinese herbal medicine (CHM) has gained widespread application in the treatment of metabolic diseases, largely due to its clinical efficacy and safety profile. Such multi-component systems are increasingly recognized for their ability to simultaneously regulate uric acid metabolism, oxidative stress, and inflammation, thereby providing a more integrated therapeutic strategy [26,27,28]. Current studies have indicated that Archidendrin I exhibits xanthine oxidase inhibitory activity, suggesting the potential of *Archidendron clypearia* as a novel therapeutic agent.

In this study, APE2025, a polyphenol-rich extract obtained under optimized conditions, demonstrated significant hypouricemic activity across in vitro and in vivo models. Mechanistically, APE2025 simultaneously inhibited key enzymes involved in uric acid production and modulated renal urate transporters. This dual regulatory effect suggests that APE2025 acts on both the generation and elimination pathways of uric acid, thereby contributing to a more comprehensive restoration of urate homeostasis. Such coordinated regulation represents a typical multi-target pharmacological profile and may explain its consistent efficacy across experimental models. Notably, the biological activity observed in APE2025 can be associated with its optimized chemical composition, indicating that compositional enrichment achieved through extraction directly contributes to pharmacological efficacy.

At the molecular level, the inhibition of XOD by APE2025 may be associated with the structural characteristics of its polyphenolic constituents. Hydroxyl-rich polyphenols can interact with the active site of XOD through hydrogen bonding and π–π interactions, leading to competitive or mixed-type inhibition [29]. In addition, their antioxidant properties may indirectly suppress XOD activity by modulating the redox environment required for enzyme activation.

Regarding urate transport, the observed downregulation of URAT1 and GLUT9 and upregulation of ABCG2 suggest that APE2025 influences key pathways governing uric acid reabsorption and excretion. This modulation may not solely result from direct interaction with transporters but could involve upstream regulation via oxidative stress and inflammatory signaling pathways, which are known to affect transporter expression [30]. The coordinated regulation of these transporters facilitates reduced urate reabsorption and enhanced excretion, complementing the inhibition of uric acid production. Together, these findings support a coherent relationship in which optimized extraction conditions lead to compositional changes that subsequently drive multi-target pharmacological effects.

In addition to its urate-lowering effects, APE2025 exhibited protective effects on renal and hepatic tissues. These findings indicate that its pharmacological activity extends beyond uric acid regulation and may involve attenuation of tissue injury associated with hyperuricemia. This organ-protective effect may be related to the antioxidant and anti-inflammatory properties of polyphenols, further supporting its therapeutic potential.

It is important to note that the bioavailability and bioaccessibility of polyphenolic compounds may influence their in vivo efficacy. Polyphenols are generally characterized by limited absorption and extensive metabolism. However, their metabolites may retain biological activity, and synergistic interactions among multiple constituents may compensate for low systemic concentrations [31]. In addition, local effects in the gastrointestinal tract or kidney may also contribute to their overall pharmacological action. Therefore, further studies on pharmacokinetics, metabolic pathways, and bioavailability are required to better elucidate the in vivo behavior of APE2025.

To our knowledge, this is the first study to report the anti-hyperuricemic activity of *Archidendron clypearia* polyphenols extract and to elucidate its dual mechanism involving both synthesis inhibition and excretion promotion. The findings provide a scientific basis for developing APE2025 as a natural multi-target agent against hyperuricemia. However, several limitations of the present work should be acknowledged. First, the precise active constituents responsible for the observed effects remain to be fully characterized. Second, as this study employed an acute hyperuricemia model, the long-term efficacy and safety of APE2025 in chronic settings require further investigation. In addition, the lack of pharmacokinetic data limits a comprehensive understanding of the relationship between exposure and efficacy. Future studies integrating compound isolation, target validation, and in vivo pharmacokinetics are warranted.

In summary, through an innovative extraction process, this study produced for the first time a standardized polyphenol extract from *Archidendron clypearia* enriched in Archidendrin I. It systematically elucidated the pharmacological basis for its dual-channel action in lowering uric acid and protecting renal function. This work highlights the importance of activity-guided extraction strategies and supports the potential of multi-component natural products as integrated therapeutic agents for metabolic disorders.

## 4. Materials and Methods

### 4.1. Chemicals and Reagents

Gallic acid standard, Folin–Ciocalteu reagent, xanthine and xanthine oxidase (XO) were purchased from Macklin (Shanghai, China). Phosphoric acid (HPLC grade) and Hypoxanthine (HX) were obtained from Aladdin (Shanghai, China). Acetonitrile (HPLC grade) was purchased from Osenbaker (Tianjin, China). Anhydrous ethanol and methanol was supplied by Energy Chemical (Shanghai, China). Sodium carbonate was obtained from Tianjin Zhiyuan Chemical Reagent Co., Ltd. (Tianjin, China). Potassium oxonate (PO) and adenosine were provided by Yuanye (Shanghai, China). Assay kits for detecting UA (C012), adenosine deaminase (ADA, A048) and superoxide dismutase (SOD, A001) were obtained from Nanjing Jiancheng Biotechnology Institute (Nanjing, China). Assay kits for detecting Xanthine oxidase (XOD) were obtained from Solarbio (Beijing, China). Sodium hydroxide and hydrochloric acid were purchased from Guangzhou brand (Guangzhou, China). CCK-8 assay kit was provided by Dojindo (Kumamoto, Japan). Phosphate-Buffered Saline was purchased from Procell (Wuhan, China). A Millipore ultrapure water machine (Millipore, Molsheim, France) prepared pure ultra-pure water.

Archidendrin I (purity ≥ 98%) was isolated from *A. clypearia* according to a previously established procedure with minor modifications. Briefly, the dried and powdered plant material (leaves and branches, 5 kg) was extracted three times with 95% ethanol (20 L each) by maceration at room temperature for 1 week. The combined extracts were concentrated under reduced pressure to afford a crude ethanol extract (950 g). A portion of the extract (20 g) was subjected to macroporous adsorption resin (XAD-16N, Zhengzhou Hecheng New Material Technology Co., Ltd., Zhengzhou, China) column chromatography and eluted with a gradient of ethanol–water (4:6 → 7:3, *v*/*v*) to yield four fractions (X1–X4). Fraction X4 was further separated by reversed-phase C18 silica gel (YMC Co., Ltd., Kyoto, Japan) column chromatography using a methanol–water gradient (8:2 → 7:3, *v*/*v*), affording subfractions X4C1 and X4C2. Subfraction X4C2 was further purified by Sephadex LH-20 (Shanghai Yuanye Biotechnology Co., Ltd., Shanghai, China), Chinacolumn chromatography with methanol as the eluent to yield Archidendrin I (2 g).

### 4.2. Preparation of Raw Material

The experimental samples comprised dried twigs and leaves of *Archidendron clypearia*, collected from Maogang Village, Zhongluotan Town, Baiyun District, Guangzhou. The samples were authenticated by Professor Yang Depo from the School of Pharmaceutical Sciences, Sun Yat-sen University, as the dried branches and leaves of *Archidendron clypearia* (Fabaceae). After removal of impurities, the samples were pulverized, sieved through a 50-mesh sieve, and stored in a desiccator for subsequent use.

### 4.3. Measurement Method of Indicator Components

#### 4.3.1. Folin–Ciocalteu Method Determination of Total Polyphenol Content (TPC)

According to the method reported in the literature, the method adopted was slightly modified [32]. First, 10 µL of standard or sample solution at different concentrations was added to 1.5 mL centrifuge tubes, followed by the addition of 45 µL of Folin–Ciocalteu reagent. After being mixed and standing for 5 min, 225 µL of 10% sodium carbonate solution and 470 µL of distilled water were added. The mixture was homogenized and kept in the dark for 1 h, then centrifuged (8000 rpm, 5 min). The supernatant was collected, and the absorbance was measured at 765 nm. TPC was described as milligrams of gallic acid per gram of powder.

#### 4.3.2. HPLC-DAD Determination of Archidendrin I Content (ADC)

Chromatographic analysis was performed using a Shimadzu LC-20AB high-performance liquid chromatography system (Nakagyo-ku, Japan) equipped with a C18 reversed-phase column (Eclipse XDB 80 Å, 4.6 × 250 mm, 5 µm). The chromatographic conditions were as follows: flow rate, 1 mL/min; column temperature, 40 °C; detection wavelength, 278 nm; injection volume, 10 µL. The mobile phase consisted of 0.2% phosphoric acid aqueous solution (A) and acetonitrile (B) with the following gradient elution program: 0 min–15 min, 95–75% A; 15 min–25 min, 75% A; 25 min–27 min, 75–70% A; 27 min–30 min, 70% A. The content of Archidendrin I (ADC) was expressed as milligrams of Archidendrin I per gram of powder.

### 4.4. Optimization of Processes

#### 4.4.1. Extraction Procedure

Extraction was performed using a PS-60AL (40 kHz) digital ultrasonic cleaner (Layton, Suzhou, China). Approximately 0.1 g of the herbal powder was placed in a 5 mL centrifuge tube, and ethanol was added according to the set material-to-liquid ratio. Ultrasonic extraction was carried out at a specified ultrasonic power and for a defined duration. After extraction, the mixture was centrifuged and filtered, and the supernatant was collected for subsequent steps.

#### 4.4.2. Single-Factor Experimental Design

The default extraction conditions were as follows: ethanol concentration, 50%; liquid-to-material ratio, 30; ultrasonic time, 30 min; and ultrasonic power, 100 W. The other conditions were kept constant when one of the factors was examined. The effects of four factors—ethanol concentration (10%, 30%, 50%, 70%, and 90%), liquid-to-material ratio (10, 20, 30, 40, and 50), sonication time (5, 10, 20, 30, 40, and 50 min), and extraction cycles (1, 2, and 3)—on the yield of total polyphenols and Archidendrin I from twigs and leaves of *Archidendron clypearia* were investigated.

#### 4.4.3. Response Surface Design

A Box–Behnken (B-B) design was used in optimizing the *Archidendron clypearia* extraction parameters. Different factors with high and low levels are shown in Table S3. A three-factor three-level experimental design was performed. Ethanol concentration (A), liquid-to-material ratio (B), and sonication time (C) were selected as investigated factors. A high level was represented by 1, whereas a low level was represented by −1. TPC and ADC were used as evaluation indexes.

#### 4.4.4. Calculation of Comprehensive Evaluation Value (CEV) by Entropy Weighting Method (EWM)

The entropy weight method (EWM) is an objective approach for assigning weights to evaluation indicators. In this study, the measured values of each index obtained from the Box–Behnken (B-B) experimental design were first normalized using the following formula: normalized score = (measured value − minimum value)/(maximum value − minimum value). This step eliminated scale- and magnitude-related discrepancies among different indicators. Subsequently, the weight coefficients for each index were determined via EWM based on their normalized data. Finally, a comprehensive evaluation value (CEV) was calculated by integrating the weighted index scores.

### 4.5. Preparation and Qualitative Analysis of Polyphenolic Extracts from Archidendron clypearia (APE2025)

Based on the optimized parameters, a measured quantity of *Archidendron clypearia* powder was extracted with 50% (*v*/*v*) ethanol at a liquid-to-material ratio of 30 using an ultrasonic cleaner for 31 min. A second extraction was carried out under the same ethanol concentration. The combined filtrates were concentrated under reduced pressure until the odor of ethanol disappeared and then freeze-dried to obtain the polyphenolic extracts from *Archidendron clypearia* (APE2025). The resulting powder was transferred into a sealed centrifuge tube, sealed with parafilm, and stored at 4 °C for further use. The total polyphenol content was quantified by Folin–Ciocalteu method, and the Archidendrin I (7-O-galloyltricetiflavan) content was quantified by HPLC-DAD determination.

Polyphenol extracts were diluted fivefold with 50% (*v*/*v*) ethanol and subsequently filtered through a 0.22 µm membrane. Chromatographic separation was conducted on an Acquity UPLC HSS T3 column (2.1 mm × 100 mm, 1.8 µm). Mass spectrometric analysis was performed using an AB X500R QTOF instrument (SCIEX, Framingham, MA, USA). The column temperature was set at 30 °C, with a flow rate of 0.3 mL/min and an injection volume of 5 µL. The mobile phases consisted of solvent A (0.1% (*v*/*v*) formic acid in water) and solvent B (methanol). The gradient elution program was as follows: 0–2 min, 6–17% B; 2–10 min, 17–23% B; 10–15 min, 23–26% B; 15–20 min, 26–30% B; 20–25 min, 30–32% B; 25–30 min, 32% B; 30–40 min, 32–38% B; 40–50 min, 38–48% B. MS/MS conditions were as follows: capillary temperature, 550 °C; ion spray voltage, 5.5 kV; declustering potential, 80 V; collision energy, 10 V; full scan mass range, m/z 500–1000; all ions were monitored in both positive and negative ionization modes.

### 4.6. Xanthine Oxidase Inhibitory Activity

Xanthine oxidase (XOD) catalyzes the conversion of xanthine to uric acid, which absorbs at 290 nm. XOD inhibitory activity was assessed using an in vitro enzymatic assay. Briefly, 50 µL of sample (or PBS control) and 50 µL of XOD working solution (75 U/L) were incubated at 37 °C for 15 min. The reaction was initiated by adding 50 µL of xanthine (150 µM), incubated for 30 min at 37 °C, and terminated with 50 µL of 1 M HCl. Absorbance was measured at 290 nm. Allopurinol served as the positive control. The substrate (15 mM) and enzyme (500 U/mL) stock solutions were prepared in phosphate buffer (pH 7.4) and diluted to working concentrations prior to use. The enzymatic reaction is represented as follows:

Xanthine + H_2_O + O_2_ → XOD → Uric acid + H_2_O_2_

### 4.7. Cell Culture and Model Construction

HK-2 cells (Chinese Academy of Sciences) were cultured in DMEM/F12 with 10% FBS at 37 °C, 5% CO_2_. The method of model construction was obtained from the literature and slightly modified [33]. Cells were planted in 24-well plates (8 × 10^4^ cells/well) for 24 h, then treated with APE2025 (6.25, 12.5, 25 µg/mL) in serum-free medium for 24 h. After washing with PBS, cells (except control) were exposed to adenosine (2.5 mmol/L) for 30 h, followed by xanthine oxidase (0.5 U/mL) for 8 h to induce hyperuricemia. Control cells received XOD only without adenosine. Supernatants were collected for uric acid measurement.

### 4.8. Cell Viability and Uric Acid Quantification

Polyphenolic extract was dissolved in DMSO and diluted with serum-free DMEM-F12 medium to final concentrations of 50, 25, 12.5, and 6.25 µg/mL. The final concentration of DMSO was below 0.1%. Following the previously described modeling procedure, the cells were treated accordingly. After modeling, the supernatant was removed, and 10 µL of CCK-8 solution was added to each well under light-protected conditions. The plates were further incubated for 3 h, after which the absorbance was measured at 450 nm using a microplate reader (BMG LABTECH, Ortenberg, Germany). Uric acid quantitative detection was performed using a Uric Acid Assay Kit (Jiancheng, Nanjing, China).

### 4.9. Animals and Treatment

Male Kunming mice aged 6–8 weeks (25–30 g) were purchased from the Laboratory Animal Center of Sun Yat-sen University (Guangzhou, China) (SYXK 2023-0112). Animals were acclimated for 1 week before the experiments. All animals were allowed to eat a standard diet and drink ad libitum and adapted to the specific pathogen-free (SPF) conditions at 25  ±  2 °C, humidity 60  ±  5%, with a fixed 12 h artificial light period. The order of treatment administration and outcome measurement was randomized across groups. Animals were housed in a single ventilated rack with cages arranged in a randomized layout. All behavioral tests and sample collections were performed at the same time of day (9:00–11:00 AM) to avoid circadian effects. The study was guided and approved by the Animal Ethics Committee of Sun Yat-sen University (SYSU-IACUC-2025-002124).

The sample size was determined based on previous similar pharmacological studies and our preliminary experiments. Sixty male Kunming mice were randomly assigned to six groups (*n* = 10): control, model, allopurinol (5 mg/kg), and APE2025 (50, 100, 200 mg/kg). Hyperuricemia was induced by daily intragastric administration of potassium oxonate (PO, 250 mg/kg) and hypoxanthine (HX, 150 mg/kg) suspended in 0.5% CMC-Na. One hour later, APE2025 or allopurinol (dissolved in 10% DMSO/40% PEG300/5% Tween 80/45% saline) was administered intragastrically. Control mice received vehicles only. Treatments were repeated once daily for 9 consecutive days. Body weight was recorded daily. On day 9, mice were anesthetized with 2% isoflurane; blood, kidneys, and liver were collected. Serum was separated by centrifugation (3000 rpm, 10 min, 4 °C) and stored at −80 °C. Tissues were divided: one part fixed in 4% paraformaldehyde for histopathology, the other stored at −80 °C for biochemical analysis. The kidney coefficient (kidney weight/body weight) was calculated. Animals with body weight deviations exceeding ±20% of the group average at baseline, as well as those that died during the experimental period, were excluded from the analysis. All animals were included in the final analysis, and no data points were excluded.

### 4.10. Serum and Liver Biochemical Analysis

The levels of UA, ADA, and SOD in the serum were detected by using commercial detection kits (Jiancheng Bioengineering Institute, Nanjing, China) according to the instructions of manufacturers. And XOD in the liver was detected by commercial detection kits (Solarbio, China). The levels of blood urea nitrogen (BUN) and creatinine (CRE) were measured using an automatic biochemical analyzer at the Laboratory Animal Center of Sun Yat-sen University (Guangzhou, China).

### 4.11. Hematoxylin and Eosin (H&E) Staining

Paraffinized sections (5 µm thickness) were used for H&E staining and subsequent pathological analysis according to the protocols previously described.

### 4.12. RNA Isolation and Real-Time PCR Analysis

Total RNA was extracted from kidney tissues (20 mg) using Trizol reagent. Following chloroform addition and centrifugation (12,000 *g*, 15 min, 4 °C), the aqueous phase was collected. RNA was precipitated with isopropanol, pelleted by centrifugation (12,000 *g*, 10 min, 4 °C), washed twice with 75% ethanol, and dissolved in DEPC-treated water. RNA concentration was measured using a Nanodrop 2000 (Wilmington, DE, USA).

RNA (1 µg) was reverse transcribed to cDNA using a Color Reverse Transcription Kit (EZ Bioscience, Roseville, MN, USA). Quantitative real-time PCR was performed with Color SYBR Green qPCR Master Mix (EZ Bioscience) on a Roche LightCycler system. The primer sequences for the target genes are listed in Table S4. Cycling conditions: 95 °C for 30 s, followed by 40 cycles of 95 °C for 10 s and 60 °C for 30 s. Relative mRNA expression was calculated using the 2^−ΔΔCt^ method with β-actin as the housekeeping gene.

### 4.13. Statistical Analysis

All data were presented as mean ± standard deviation of three replicates, and data analyses were performed using GraphPad Prism 9.0.0 and IBM SPSS Statistics 25.0 software. Comparisons between two groups were analyzed using Student’s *t*-test, while comparisons among multiple groups were performed using one-way analysis of variance (ANOVA), followed by Tukey’s post hoc test. The normality and homogeneity of variance assumptions were tested and found to be satisfied. When these assumptions were violated, the Kruskal–Wallis test followed by Dunn’s post hoc test was applied.

## 5. Conclusions

In conclusion, an environmentally friendly and efficient method has been established to maximize the extraction of polyphenols and Archidendrin I from *Archidendron clypearia* using ethanol-assisted ultrasonication. Through single-factor experiments and response surface optimization, improved extraction conditions were determined, including ethanol concentration, material-to-liquid ratio, and extraction time. The entropy weight method was applied to identify the optimal process parameters. Under the optimal conditions, the yields of total polyphenol content (TPC) and Archidendrin I content (ADC) reached 175 mg/g DW and 80.34 mg/g DW, respectively. Moreover, the *Archidendron clypearia* polyphenol extract obtained through this process exhibited significant uric acid-lowering effects both in vitro and in vivo. Its mechanism involves dual-channel regulation: inhibiting xanthine oxidase activity, downregulating renal uric acid reabsorption transporters (URAT1/GLUT9), and upregulating the excretion transporter ABCG2 while also ameliorating kidney and liver tissue damage induced by hyperuricemia. This study not only provides an optimized extraction strategy for *A. clypearia* extracts but also lays a scientific foundation for the development of multi-target, naturally sourced anti-hyperuricemic agents.

## Figures and Tables

**Table 1:** Box–Behnken Response Surface Test Design and Results.

Run	A(%)	B	C (min)	CEV
1	−1	1	0	120.637
2	0	0	0	128.434
3	1	0	−1	120.065
4	0	−1	1	122.368
5	1	−1	0	120.784
6	0	0	0	129.525
7	0	0	0	124.837
8	0	0	0	127.655
9	0	1	−1	120.499
10	−1	0	−1	113.115
11	1	1	0	124.642
12	−1	−1	0	119.661
13	0	0	0	124.975
14	0	−1	−1	114.699
15	0	1	1	120.791
16	−1	0	1	120.516
17	1	0	1	122.116

**Table 2:** ANOVA for Response Surface Quadratic model.

Source	Sum of Squares	df	Mean Square	F-Value	*p*-Value	
Model	285.26	9	31.70	11.44	0.0020	significant
A—ethanol concentration	23.39	1	23.39	8.44	0.0228	significant
B—liquid-to-material ratio	10.25	1	10.25	3.70	0.0958	
C—sonication time	37.90	1	37.90	13.68	0.0077	significant
AB	2.08	1	2.08	0.7490	0.4155	
AC	7.16	1	7.16	2.58	0.1521	
BC	13.60	1	13.60	4.91	0.0623	
A^2^	41.65	1	41.65	15.03	0.0061	significant
B^2^	26.51	1	26.51	9.57	0.0175	significant
C^2^	104.73	1	104.73	37.79	0.0005	significant
Residual	19.40	7	2.77			
Lack of Fit	1.79	3	0.5963	0.1355	0.9339	not significant
Pure Error	17.61	4	4.40			
Cor Total	304.66	16				

**Table 3:** Compounds identified from APE2025 in positive ion mode.

No.	RT (min)	Formula	Mass	Error	Fragment	Compound
1	2.31	C7H605	171.0284	0.8	153.0176, 126.0312	Gallic acid (**1**)
2	5.38	C14H20N2O3	265.1539	3.1	177.0544, 145.0281	Feruloylputrescine
3	4.74	C15H1407	307.0806	0	139.0389	epigallocatechin
4	10.22	C9H10O5	199.0594	3.4	153.0180, 109.0283, 53.0384	Ethyl gallate
5	12.45	C22H18O10	443.0969	1	165.0547, 153.0182, 139.0391	7-O-galloylcatechin
6	20.65	C22H18O10	443.0972	0.4	291.0854, 165.0544	Archidendrin I (7-O-Galloyltricetiflavan) (**2**)
7	23.88	C29H22O14	595.1086	0.1	443.0977, 153.0181	7,4′-di-O-galloyltricetiflavan (**3**)
8	26.24	C21H20O11	449.1091	1.4	303.0496	Quercitrin (**4**)
9	35.10	C29H22O14	595.1088	0.3	443.0966, 153.0185	7,3′-di-O-galloyltricetiflavan (**5**)
10	39.39	C15H10O6	287.0551	0.4	153.0180	Luteolin

**Table 4:** Compounds identified from APE2025 in negative ion mode.

No.	RT (min)	Formula	Mass	Error	Fragment	Compound
1	0.94	C12H22011	341.108	1.3	59.0136, 89.0240, 161.0459	Alpha-Lactose
2	1.42	C6H807	191.0195	1.2	111.0093	Isocitric acid
3	2.78	C7H605	169.0137	3.9	126.0274, 80.0224	Gallic acid (**1**)
4	5.91	C15H1407	305.0659	0.3	125.0242	epigallocatechin
5	10.86	C22H18O11	457.0762	0.7	305.0653, 125.0241	3,5,3′,4′,5′-pentahydroxyflavan-7-gallate
6	11.37	C9H10O5	197.0446	0.3	170.0170, 125.0204, 78.0109	Ethyl gallate
7	13.87	C22H18O10	441.0814	0.4	137.0244, 151.0594	7-O-galloylcatechin
8	14.86	C22H18O11	457.0761	0	305.0652, 125.0242, 137.0244	Epigallocatechin-7-O-gallate
9	17.05	C21H20O13	479.0821	0	316.022	Myricetin-3-galactoside
10	20.07	C21H20O12	463.0875	0.6	317.0253	Myricitrin
11	21.37	C21H20O12	463.0875	0.3	300.0261	Isoquercitrin
12	21.53	C22H18O10	441.0804	0.9	137.0241, 151.0388	Archidendrin I(7-O-Galloyltricetiflavan) (**2**)
13	22.19	C21H20O12	463.0874	0	300.0267	Hyperoside
14	23.65	C21H22O11	449.1092	0.7	287.0559, 151.0039	Eriodictyol 3-O-glucoside
15	23.91	C20H18O11	433.0772	0.2	300.028	Avicularin
16	24.68	C29H22O14	593.0919	0.6	442.0835, 137.0237	7,4′-di-O-galloyltricetiflavan (**3**)
17	25.46	C21H20O11	447.0932	0.1	284.0379	Astragalin
18	27.14	C21H20O11	447.0925	0.7	300.0259, 255.0290, 151.0036	Quercitrin (**4**)
19	35.64	C21H20O10	431.0985	0.2	284.0332	Kaempferol-7-O-alpha-L-rhamnoside
20	35.82	C29H22O14	593.0924	0.2	442.0847, 137.0240	7,3′-di-O-galloyltricetiflavan (**5**)
21	40.15	C15H10O6	285.0404	0.3	133.0295	Luteolin
22	45.04	C43H79N7O9	836.5857	0.5	790.5781	halovir B

## Data Availability

The raw data supporting the conclusions of this article will be made available by the authors on request.

## Supplementary Materials

The following supporting information can be downloaded at: https://www.mdpi.com/article/10.3390/molecules31091451/s1, Figure S1: Linear fitting curve of gallic acid concentration and absorbance. Figure S2: Linear fitting curve of archidendrin I concentration and peak area. Figure S3. HPLC chromatograms of Archidendrin I and APE2025. Table S1: Results of total phenolic compounds recovery rate. Table S2: Results of Archidendrin I recovery rate. Table S3: Factors and levels of the Box–Behnken design. Table S4: Sequences of primers for real-time PCR.

## Abbreviations

The following abbreviations are used in this manuscript:

APE2025	*Archidendron clypearia* polyphenols extract
HUA	Hyperuricemia
UA	Uric Acid
XOD	Xanthine Oxidase
GLUT9	Glucose Transporter 9
URAT1	Urate Transporter 1
ABCG2	ATP-Binding Cassette Transporter G2
TPC	Total Polyphenol Content
ADC	Archidendrin I Content
CEV	Comprehensive Evaluation Value
HPLC	High-Performance Liquid Chromatography
IC_50_	Half-Maximal Inhibitory Concentration
PBS	Phosphate-Buffered Saline
FBS	Fetal Bovine Serum
CMC-Na	Carboxymethyl Cellulose Sodium
PEG300	Polyethylene Glycol 300
BUN	Blood Urea Nitrogen
CRE	Creatinine
ADA	Adenosine Deaminase
SOD	Superoxide Dismutase
H&E	Hematoxylin and Eosin
qPCR	Quantitative Real-Time Polymerase Chain Reaction
CHM	Chinese Herbal Medicine
RSM	Response Surface Methodology
EWM	Entropy Weight Method
