# Supplementary material for: Polyphenol-Rich Extract from Archidendron clypearia: Optimization, Characterization, and Hypouricemic Activity
Source: Molecules. 2026 Apr 27;31(9):1451. doi: 10.3390/molecules31091451 (PMC13165188; doi:10.3390/molecules31091451)
Supplement: Supplementary file 1 [file molecules-31-01451-s001.zip › molecules-4258534-supplementary.pdf]

Polyphenol-Rich Extract from Archidendron clypearia: Optimization, Characterization, and Hypouricemic Activity

Danna Yan1, Ziyan Hong1 , Zhimin Zhao1 , Wenzhe Yang2 , Depo Yang1,2*

1 School of Pharmaceutical Sciences, Sun Yat-Sen University, Guangzhou 510006, China;

yandn@mail2.sysu.edu.cn(D.Y.),hongziyansysu@163.com(Z.H.),zhaozhm2@mail.sysu.edu.cn(Z.Z.)

2 Zhongshan Unicare Natural Medicine Co., Ltd.， Zhongshan 528437, China;fabrice0409@163.com

* Correspondence: lssydp@mail.sysu.edu.cn; Tel.: 13640706628

1. Total Polyphenols (Folin–Ciocalteu Method)

1.1 Calibration curve

The fitting equation of gallic acid was y = 0.8191x + 0.0296 (R^2 = 0.9976). Absorbance was used as the vertical coordinate, and gallic acid concentration was used as the horizontal coordinate. Gallic acid had a good linear correlation with absorbance in a concentration range of 0.2–1.2 mg/mL. (Fig.S1)

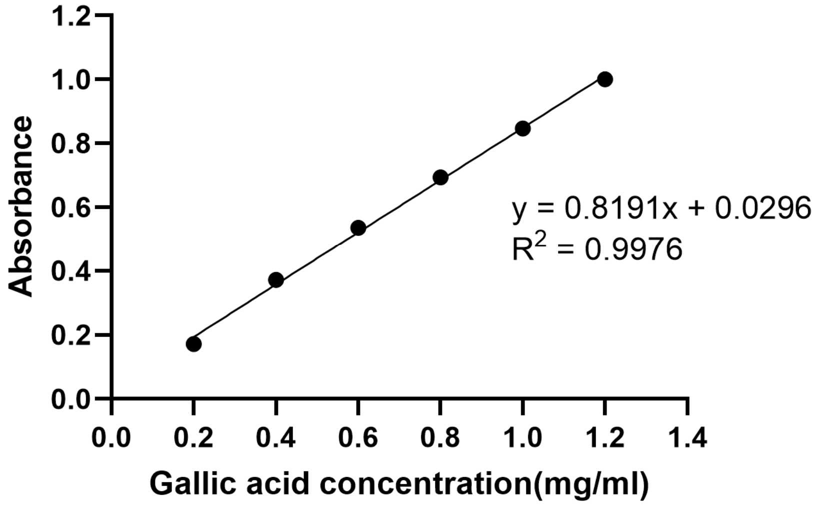

Figure S1. Linear fitting curve of gallic acid concentration and absorbance.

1.2 Precision

The precision was evaluated by measuring the absorbance of a gallic acid standard solution (0.8 mg/mL) six times consecutively. The relative standard deviation (RSD) of absorbance was 0.06%, indicating good instrument precision.

1.3 Repeatability

Six independently prepared sample solutions were analyzed. The mean total polyphenol content was 191.12 mg/g with an RSD of 2.31%, demonstrating acceptable repeatability.

1.4 Stability

The same sample solution was analyzed at 0, 10, 20, 30, 40, and 60 min after color development. The RSD of absorbance over 60 min was 0.19%, indicating good stability within this period.

1.5 Recovery

Recovery was assessed by spiking known amounts of gallic acid into samples at low, medium,

and high concentration levels (nine determinations). The average recovery was 99.37% with an RSD of 2.97%, confirming the accuracy of the method.

Table S1. Results of total phenolic compounds recovery rate.

**Sample**	**Sample Weight (g)**	**Content in Sample (mg)**	**Spiked Amount (mg)**	**Measured Amount (mg)**	**Recovery (%)**	**Mean Recovery (%)**	**RSD (%)**
Low-1	0.2506	1.5834	0.6657	2.2744	103.80		
Low-2	0.2506	1.5834	0.6657	2.2679	102.82		
Low-3	0.2510	1.5859	0.6657	2.2654	102.07		
Medium-1	0.2507	1.5840	1.2184	2.8110	100.70		
Medium-2	0.2506	1.5834	1.2184	2.8170	101.25	100.08	2.75
Medium-3	0.2498	1.5784	1.2184	2.7871	99.21		
High-1	0.2507	1.5840	1.8276	3.3398	96.07		
High-2	0.2506	1.5834	1.8276	3.3436	96.31		
High-3	0.2505	1.5828	1.8276	3.3822	98.46		

2. Archidendrin I (by HPLC)

2.1 Calibration curve

The standard curve regression equation of archidendrin I was obtained as y = 9E+06x - 72348 (R^2 = 0.9996), in which the horizontal coordinate was archidendrin I concentration and the vertical coordinate was peak area. The equation showed that archidendrin I showed a good linear relationship with absorbance in a concentration range of 0.01–1.02 mg/mL (Figure S2). The HPLC chromatograms (Figure S3) demonstrated clear separation and stable retention times of the target compounds, confirming the suitability of the analytical system for subsequent evaluation of extraction efficiency.

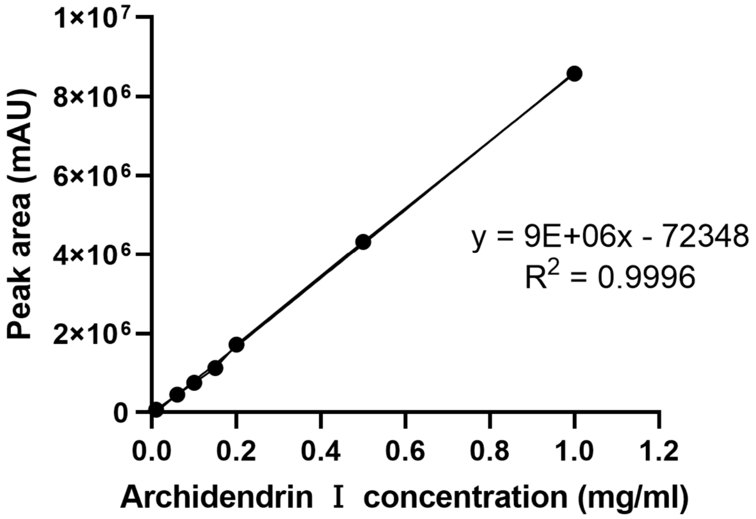

Figure S2. Linear fitting curve of archidendrin I concentration and peak area.

2.2 Precision

The mixed standard solution was injected six times consecutively. The RSD of Archidendrin I peak areas was 0.50%, indicating excellent instrument precision.

2.3 Repeatability

Six independently prepared sample solutions were analyzed. The mean Archidendrin I content was 54.24 mg/g with an RSD of 1.70%, demonstrating good repeatability.

2.4 Stability

The same sample solution was analyzed at 0, 4, 8, 12, 16, and 24 h. The RSD of Archidendrin I peak areas was 2.04%, confirming stability within 24 h.

2.5 Recovery

Recovery was determined by spiking known amounts of Archidendrin I standard into samples at low, medium, and high concentration levels (nine determinations). The average recovery was 99.27% with an RSD of 2.56%, verifying the accuracy and reliability of the method.

Table S2. Results of Archidendrin I recovery rate.

**Sample**	**Sample Weight (g)**	**Content in Sample (mg)**	**Spiked Amount (mg)**	**Measured Amount (mg)**	**Recovery (%)**	**Mean Recovery (%)**	**RSD (%)**
Low-1	0.2517	7.5034	3.7529	11.1563	97.34	99.27	2.56
Low-2	0.2500	7.4527	3.7529	11.2842	102.10		
Low-3	0.2502	7.4587	3.7529	11.0352	95.30		
Medium-1	0.2505	7.4676	7.5058	14.9910	100.24		

Sample	Sample Weight (g)	Content in Sample (mg)	Spiked Amount (mg)	Measured Amount (mg)	Recovery (%)	Mean Recovery (%)	RSD (%)
Medium-2	0.2507	7.4736	7.5058	14.9710	99.89		
Medium-3	0.2503	7.4616	7.5058	15.0326	100.87		
High-1	0.2503	7.4616	11.2586	18.2839	96.12		
High-2	0.2504	7.4646	11.2586	18.6174	99.06		
High-3	0.2500	7.4527	11.2586	18.9933	102.50		

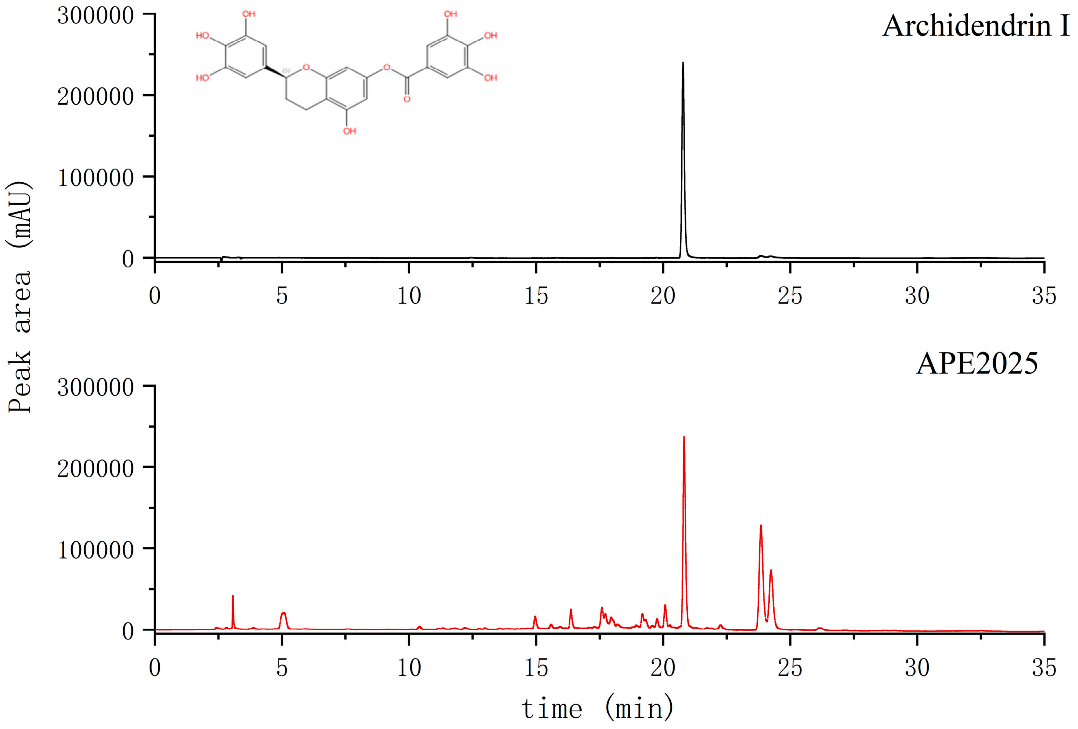

Figure S3. HPLC chromatograms of Archidendrin I and APE2025.

Table S3. Factors and levels of the Box–Behnken design.

Factor	Level		
	-1	0	1
A: Ethanol concentration (%)	40	50	60
B: Liquid-to-material ratio	20	30	40
C: Sonication time(min)	20	30	40

Table S4. Sequences of primers for real-time PCR.

Gene	Forward primer (5′-3′)	Reverse primer (5′-3′)
GLUT9	GGATTCCAGGGAGCTTGCTTT	GAGCGAGAAGGACCATTTCTTTG
URAT1	GCTATCTTCTGGTGTCCGTGT	ATGTCCACTCCATCAAGAGACTG
ABCG2	CCTACAACAACCCTGCGGAT	GGCTCTTCAGTCTTGTTTGCTTC
β-actin	AGTGTGACGTTGACATCCGT	ATCCGCCTGACAATGACTCGA
